# Transcriptomic profile of leg muscle during early growth and development in Haiyang yellow chicken

**DOI:** 10.5194/aab-64-405-2021

**Published:** 2021-09-20

**Authors:** Xuemei Yin, Yulin Wu, Shanshan Zhang, Tao Zhang, Genxi Zhang, Jinyu Wang

**Affiliations:** 1 School of Marine and Bioengineering, YanCheng Institute of Technology, Yancheng, China; 2 College of Animal Science and Technology, Yangzhou University, Yangzhou, Jiangsu, China; 3 Institutes of Agricultural Science and Technology Development, Yangzhou University, Yangzhou, China

## Abstract

Skeletal muscle growth and development from embryo to adult consists of a series of carefully regulated changes in gene expression. This study aimed to identify candidate genes involved in chicken growth and development and to investigate the potential regulatory mechanisms of early growth in Haiyang yellow chicken. RNA sequencing was used to compare the transcriptomes of chicken muscle tissues at four developmental stages. In total, 6150 differentially expressed genes (DEGs) (|fold change| ≥ 2; false discovery rate (FDR) ≤ 0.05) were detected by pairwise comparison in female chickens. Functional analysis showed that the DEGs were mainly involved in the processes of muscle growth and development and cell differentiation. Many of the DEGs, such as *MSTN*, *MYOD1*, *MYF6*, *MYF5*, and *IGF1*, were related to chicken growth and development. The Kyoto Encyclopedia of Genes and Genomes (KEGG) pathway analysis showed that the DEGs were significantly enriched in four pathways related to growth and development: extracellular matrix (ECM)–receptor interaction, focal adhesion, tight junction, and insulin signalling pathways. A total of 42 DEGs assigned to these pathways are potential candidate genes for inducing the differences in growth among the four development stages, such as *MYH1A*, *EGF*, *MYLK2*, *MYLK4*, and *LAMB3*. This study identified a range of genes and several pathways that may be involved in regulating early growth.

## 1 Introduction

Skeletal muscle, which accounts for approximately 40 % of the body weight of mammals, is an important tissue involved in the regulation of metabolism, locomotion and strength (Frontera and Ochala, 2015). A reduction in skeletal muscle mass resulted in weakness and impaired mobility and, if severe enough, increased morbidity and mortality (Coelen et al., 2015; Szulc et al., 2010). In livestock production, skeletal muscle develops into meat, which is the primary terminal product for human consumption. Therefore, the study of muscle development in agriculturally important species is essential to achieving increased body weight and muscle mass.

The embryonic stage of animals is a key window stage for animal growth and development (Groothuis et al., 2005a, b; Cottrell and Ozanne, 2007), in which the combination of genetic material and the maternal nutritional environment determines the early growth and development of the offspring and even their phenotypic performance in adulthood (Ge et al., 2000; Zhao et al., 2001; McMillen et al., 2006). Embryonic growth is an insurmountable stage in the total growth period of poultry; the generation of muscle fibres, which are closely related to the yield and quality of poultry muscles, is nearly completed during the incubation period. It is well established that the number of muscle fibres is determined embryonically (Smith, 1963), as myoblasts, originating as somites, migrate to the appropriate site of muscle formation and then proliferate during the process of hyperplasia. These myoblasts then withdraw from the cell cycle, fuse to form multi-nucleated myotubes, and differentiate with the commencement of muscle-specific protein expression. The number of muscle fibres no longer increases after hatching, and the increase in muscle yield mainly depends on the thickening and lengthening of muscle fibres. Therefore, the growth and development of poultry embryonic muscle has a considerable effect on the meat-producing ability of adult animals.

In this study, Haiyang yellow chicken, a nationally cultivated quality broiler breed in China, was used to study transcriptomic changes during different growth stages. Jinghai yellow chicken, which is one of the parents of Haiyang yellow chicken, was independently cultivated in the previous period. We have successfully introduced the characteristics of Jinghai yellow chicken, including excellent meat quality, high reproductive performance, and strong resistance, into the matching line of Haiyang yellow chicken. Therefore, Haiyang yellow chicken can be listed on the market at 70 d old; the weight of a rooster can reach 2.1 kg and that of a hen can reach 1.6 kg. Haiyang yellow chickens exhibit excellent meat quality and high adaptability, overcoming the bottleneck of the breeding rate of yellow feather broilers and improving the core technical level of matching lines in China. Chicken growth, an important economic trait, is controlled by multiple genes. Many studies identified genetic factors affecting growth. Candidate genes and quantitative trait loci (QTLs), such as *GH*, *IGFBP2* and *GHSR*, have been identified (Niarami et al., 2014; Ahsan et al., 2013). Xue et al. (2017) used RNA-Seq to compare the transcriptomes of chicken muscle tissues at three developmental stages during early growth. In total, 978 differentially expressed genes (DEGs) were detected by pairwise comparison. There were 42 potential candidate DEGs that induced differences in growth among the three developmental stages (Xue et al., 2017). Therefore, in the present study, Haiyang yellow chicken at the stages of 12 d embryos (E12, pre-differentiation of skeletal muscle), 16 d embryos (E16, skeletal muscle differentiation anaphase), 1 d post-hatching (1 d, shift from myoblast-mediated growth to satellite cell-modulated growth by hypertrophy), and 10 weeks (w) (10 w, market age) were selected to study the changes in gene expression at the genome-wide level during the critical period of leg muscle growth and development and to then identify candidate genes involved in chicken growth.

Most studies on chicken growth and development conducted in the past decades have mainly focused on breast muscle (Chen et al., 2015; Piórkowska et al., 2016), whereas little information on the leg muscle transcriptome exists. The broiler industry has been very successful in selecting broilers with increased yields. However, the regulatory mechanisms underlying the growth and development of chicken leg muscle have been neglected. Therefore, the present study was carried out to study the transcriptomic changes in leg muscle during different growth stages. RNA-Seq technology and bioinformatic tools were used to investigate the major DEGs and pathways. In addition, quantitative real-time polymerase chain reaction (qPCR) experiments were performed to validate the RNA-Seq results. The results of this study are useful for understanding the mechanisms regulating the development of leg muscle and the pattern of chicken growth. The findings should provide a basis for increasing chicken leg muscle yields in the broiler industry.

## 2 Materials and methods

### 2.1 Ethics statement

All animal experiments were performed in accordance with the protocol of the Animal Use Committee of the Chinese Ministry of Agriculture and were approved by the Animal Care Committee of the Department of Animal Science and Technology, Yangzhou University. All efforts were made to minimize animal suffering.

### 2.2 Animals and tissues

The chickens used in this study were Haiyang yellow chickens, which is a breeding variety for which Jinghai yellow chicken is one of the parents. They were obtained from Jiangsu Jinghai Poultry Industry Group Co., Ltd. (Nantong city, Jiangsu province, China). Three female chickens with similar weights were selected for the E12, E16, 1 d, and 10 w age groups. Therefore, 12 female chickens from four different growth stages were used for RNA-Seq. We first used xylazine hydrochloride (SIGMA, X-1251) to anaesthetize the chickens with a dose of 8 mg/kg. When the feathers on both the wings and tails went limp and finally failed to respond to stimuli, the chickens were completely anaesthetized. The leg muscle was then immediately collected and snap-frozen in liquid nitrogen and stored at -80 ${}^{\circ }$C until RNA extraction.

### 2.3 Total RNA extraction and quality testing

Total RNA was extracted from above tissues (50–100 mg) using Trizol reagent (TakaRa Biotechnology Dalian Co., Ltd, China) according to the manufacturer's instructions. Precipitated RNA was dissolved in 30 µL RNase-free H${}_{2}$O and stored at -80 ${}^{\circ }$C. The RNA quality and quantity were assessed by agarose gel electrophoresis and Nanodrop-1000 spectrophotometer, respectively.

### 2.4 cDNA library construction and sequencing

The Oligo(dT) magnetic beads were used to purify the mRNA after the samples were tested. Subsequently, a fragmentation buffer was added to randomly fragment the mRNA, and the fragmented mRNA was used as a template to synthesize the first and second strand cDNA, respectively, and then use AMPure XP beads to purify the double-strand cDNA. The 3${}^{\prime }$ end and 5${}^{\prime }$ end refer to a deoxynucleotide chain of a DNA molecule with a 3${}^{\prime }$ end at one end and a 5${}^{\prime }$ end at the other end. In the two deoxynucleotide chains of the DNA molecule, each a skeleton of phosphate and deoxyribise, there is at one end a free phosphate group called the 5${}^{\prime }$ end and the hydroxyl group at the other called the 3${}^{\prime }$ end. The next step is to fill in the ends of the double-stranded cDNA fragments and add A to the 3${}^{\prime }$ end (the 3${}^{\prime }$ end and 5${}^{\prime }$ end refer to a deoxynucleotide chain of a DNA molecule with a 3${}^{\prime }$ end at one end and a 5${}^{\prime }$ end at the other end; for the two deoxynucleotide chains of the DNA molecule, each a skeleton of phosphate and deoxyribise, one end is a free phosphate group called the 5${}^{\prime }$ end and the hydroxyl group at the other end called the 3${}^{\prime }$ end). Finally, PCR amplification and purification of PCR products are performed to obtain the final library.

The first step is to use Qubit 2.0 for preliminary quantification, dilute the library to 1.5 ng/µL and then use Agilent 2100 to perform quality inspection on the library. After meeting the requirements, the next step is to use the RT-PCR method to detect the effective concentration of the library (the effective concentration of the library > 2 nM). The purpose is to ensure the quality of the library. After the library is qualified, the non-stop library will be pooled according to the effective concentration and target offline data volume for Illumina HiSeq sequencing.

### 2.5 Quality control and comparative analysis

The raw data (raw reads) in fastq format were first processed using in-house Perl scripts. Using this step, clean data (clean reads) were obtained by removing reads containing adapters, reads containing poly-N, and reads of low quality. At the same time, the Q20, Q30, GC (guanine and cytosine) content, and sequence duplication level of the clean data were calculated. All the downstream analyses were based on high-quality clean data.

The adaptor sequences and low-quality sequence reads were removed from the data sets. The raw sequences were transformed into clean reads after data processing. These clean reads were mapped to the reference genome sequence (Galgal4). Only reads with a perfect match or one mismatch were analysed and annotated based on the reference genome. Tophat 2 (Trapnell et al., 2012; Kim et al., 2013) was used to map the reads to the reference genome.

### 2.6 Differential expression analysis

The quantification of gene expression levels was performed as follows. The gene expression levels were estimated according to fragments per kilobase of transcript per million fragments mapped (FPKM) (Trapnell et al., 2013). Differential expression analysis between the two groups was performed using the DESeq R package (Anders et al., 2010). DESeq provides statistical routines for determining differential expression in digital gene expression data using a model based on the negative binomial distribution. The genes with a FDR ≤ 0.05 and a |fold change| ≥ 2 found by DESeq were considered differentially expressed. Fold change represents the ratio of the expression between the two groups. The resulting P values were adjusted using Benjamini and Hochberg's approach for controlling the false discovery rate.

**Table 1:** Data summary from RNA-Seq.

Sample	Raw	Clean	Clean	Q30	GC content	Total mapped
name	reads	reads	base	(%)	(%)	reads
E12-1	76 083 284	74 242 958 (97.6 %)	11.14G	86.95	51.08	62 961 871 (84.81 %)
E12-2	62 430 844	61 097 012 (97.9 %)	9.16G	91	51.22	51 452 117 (84.21 %)
E12-3	60 260 890	58 788 354 (97.6 %)	8.82G	90.14	51.71	50 893 874 (86.57 %)
E16-1	45 793 642	44 942 078 (98.1 %)	6.74G	90.63	51.38	39 444 114 (87.77 %)
E16-2	55 918 414	54 900 826 (98.2 %)	8.24G	90.46	51.11	48 153 127 (87.71 %)
E16-3	69 172 376	67 635 204 (97.8 %)	10.15G	90.67	52.5	58 406 584 (86.36 %)
1 d-1	62 058 810	60 809 868 (98.0 %)	9.12G	90.23	51.33	51 411 092 (84.54 %)
1 d-2	61 177 102	59 873 412 (98.0 %)	8.98G	90.06	51.47	50 797 742 (84.84 %)
1 d-3	65 881 404	64 654 930 (98.1 %)	9.7G	90.43	50.87	56 111 435 (86.79 %)
10 w-1	64 225 542	62 901 040 (97.9 %)	9.44G	90.92	52.05	53 426 644 (84.94 %)
10 w-2	61 984 986	60 750 640 (98.0 %)	9.11G	90.9	51.76	51 750 983 (85.19 %)
10 w-3	75 314 776	73 787 952 (98.0 %)	11.07G	91.21	51.89	62 554 090 (84.78 %)

### 2.7 GO and KEGG pathway enrichment analysis

The Gene Ontology database (Ashburner et al., 2000; Sherlock, 2009) (GO: http://geneontology. org/, last access: 9 May 2021) is a structured, standard biological annotation system built in 2000 by an organization (Gene Ontology Consortium), and it aims at establishing a standard vocabulary systematic knowledge of genes and their products. The Kyoto Encyclopedia of Genes and Genomes (KEGG) (Kanehisa et al., 2004; Teber et al., 2009) (https://www.kegg.jp/, last access: 9 May 2021) is a database resource for understanding high-level functions and utilities of the biological system, including the cell, the organism and the ecosystem, from molecular-level information, especially large-scale molecular datasets generated by genome sequencing and other high-throughput experimental technologies. All the target genes of the differentially expressed mRNA were subjected to Gene Ontology (GO) and KEGG pathway enrichment analysis by using the DAVID 6.7 functional annotation tool (Da et al., 2009) (https://david.ncifcrf.gov/, last access: 9 May 2021).

### 2.8 Validation of gene expression by quantitative real-time polymerase chain reaction analysis

Total RNA from the 12 samples used for the RNA-Seq experiment was amplified by qPCR. Single-strand cDNA was synthesized using the PrimeScript™ RT Master Mix Kit (Vazyme Biotech Co., Ltd). qPCR was performed using an Applied Biosystems 7500 real-time PCR system (Life Technologies, Gaithersburg, MD, USA) with specific primers. qPCR amplification was carried out in a 20 µL reaction volume containing 1 µL (500 ng/µL) of cDNA, 10 µL of SYBR Premix Ex Taq polymerase (2×) (Vazyme, Nanjing, China), 0.4 µL of ROX Reference Dye II (50×), 0.4 µL of the forward primer (10 mmol/L), 0.4 µL of the reverse primer (10 mmol/L), and 6.8 µL of dH${}_{2}$O. Amplification started with an initial denaturation step at 95 ${}^{\circ }$C for 30 s, followed by 40 cycles at 95 ${}^{\circ }$C for 5 s and an annealing step at 60 ${}^{\circ }$C for 34 s, at which point fluorescence was acquired. Finally, a dissociation curve to test PCR specificity was generated via one cycle at 95 ${}^{\circ }$C for 15 s followed by 60 ${}^{\circ }$C for 1 min and then ramping to 95 ${}^{\circ }$C with acquired fluorescence. Specific primers were designed based on sequences retrieved from the National Center for Biotechnology Information (NCBI) database (Table S1). Therefore, the HSP70 (Wang et al., 2015) and β-actin genes, selected as internal reference genes in the present study, were used to calculate the normalization factors based on the geometric means of these two reference gene quantities. The 2-ΔΔCt method was used to transform the data from the relative quantification step (Livak and Schmittgen, 2001).

## 3 Results

### 3.1 Blast analysis of RNA-Seq reads

We established 12 cDNA libraries, including three from each growth stage (including E12, E16, 1 d, and 10 w of age), to identify DEGs related to muscle growth and development by RNA-Seq. A total of 760, 302, and 070 raw reads were produced from the 12 cDNA libraries (Table 1). After quality control, 744, 384, and 274 clean reads (111.67 GB) were obtained. The proportion of clean reads among the raw reads of the 12 libraries ranged from 97.6 % to 98.2 %. The proportion of reads with a Phred quality value greater than 30 among the clean reads ranged from 86.95 % to 91.21 %. The average GC content of the clean reads of the 12 libraries was 51.53 %. Overall, 84.21 % to 87.77 % of the clean reads were aligned against the *Gallus gallus* reference genome.

### 3.2 Differentially expressed genes at different stages of leg muscle development

**Figure 1:**
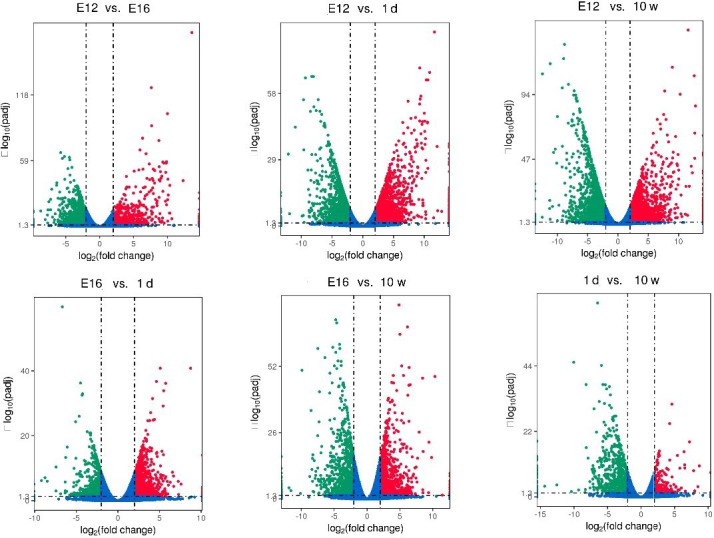
Differentially expressed genes identified between different groups of chickens.

**Figure 2:**
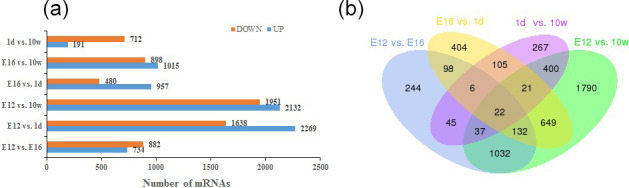
Differentially expressed genes during chicken muscle growth and development at four different stages: **(a)** numbers of upregulated and downregulated genes in female chickens; **(b)** Venn diagram of differentially expressed genes in four comparisons of female chickens (E12 vs. E16, E16 vs. 1 d, 1 d vs. 10 w, and E12 vs. 10 w).

DEGs were identified via Cuffdiff analysis (|fold change| ≥ 2; FDR ≤ 0.05). Using volcanic maps, we can see the differences in gene expression levels between different groups and the statistical significance of the differences (Fig. 1). After removing duplicate values, 6150 DEGs were detected by pairwise comparison in the female chickens (Fig. 2a and Table S2). The total number of upregulated genes was higher than the number of downregulated genes. To further analyse the interactions among the DEGs, 1616, 1437, 903, and 4083 DEGs were included in four comparisons (E12 vs. E16, E16 vs. 1 d, 1 d vs. 10 w, and E12 vs. 10 w, respectively) to construct a Venn diagram. As shown in Fig. 2b, 22 DEGs (Table S3) were common among the four comparisons.

### 3.3 GO enrichment analysis for DEGs

The DEGs were then subjected to GO analysis to reveal their functional enrichment in each comparison. These DEGs were categorized into the three main GO categories of biological process, cellular component, and molecular function. There were 147, 221, 178, 55, 45, and 233 extremely significant enriched GO terms (P<0.01) identified in E12 vs. E16, E12 vs. 1 d, E12 vs. 10 w, E16 vs. 1 d, E16 vs. 10 w, and 1 d vs. 10 w, respectively (Table S4). Many of the significantly enriched biological process terms in the comparisons of different development stages were associated with muscle growth and development, such as muscle system process, embryo development, muscle organ development, muscle tissue development, and striated muscle tissue development. A total of 23 DEGs were found for these GO terms, including genes that are well known to affect chicken growth and development, such as *MYOCD*, *MSTN*, *MYOD1*, *MYH11*, *MYF6*, *MYL4*, and *MYF5* (Table 2). The genes showing differential expression among the different stages of development were also found to be significantly enriched for terms related to the processes of muscle cell development and differentiation and striated muscle cell development and differentiation. A total of 20 DEGs were associated with these terms, and some of these genes are associated with development, such as *MYF6*, *MYOD1*, *MYH11*, and *MSTN* (Table 3). In addition, other genes, such as *MYLK2*, *MYL4*, *FGF9*, *FGF8*, *ACTA1*, *MYOM1*, and *MOMY2*, were included among these processes. These genes might be crucial for muscle development and chicken growth.

**Table 2:** Significantly enriched biological process terms involved in muscle growth and development.

Term ID	Term	Genes
GO:0003012	muscle system process	*MYLK2*, *MSTN*, *MYL1*, *MYOM1*, *MYOM2*, *MYL4*, *MYBPC1*, *MYOCD*, *MYOD1*, *MYH11*
GO:0006936	muscle contraction	*MYOM2*, *MYOCD*, *MYBPC1*, *MYOM1*, *MYLK2*, *MYL1*, *MYL4*, *MYH11*
GO:0006941	striated muscle contraction	*MYLK2*, *MYL4*
GO:0032502	developmental process	*MYLK23*, *ACTA1*, *HBEGF*, *TGFBI*, *MYF6*, *GDF5*, *MYOCD*, *MUSTN1*, *FGF8*
GO:0009790	embryo development	*MSTN*, *FGF4*, *MYF6*, *MYF5*, *FGFR2*, *FGF9*
GO:0007517	muscle organ development	*MYF5*, *MYF6*, *MYOCD*, *ACTA1*, *MYLK2*, *MSTN*, *MYOD1*, *FGF8*
GO:0061061	muscle structure development	*MSTN*, *ACTA1*, *MYLK2*, *MYOCD*, *MYF6*, *MYOD1*, *FGF10*, *FGF8*, *FGF9*, *MYH11*
GO:0060537	muscle tissue development	*FGF9*, *MYOD1*, *MYF6*, *ACTA1*, *MSTN*, *MYOCD*, *MYLK2*, *MYH11*, *FGF8*
GO:0014706	striated muscle tissue development	*MSTN*, *MYLK2*, *ACTA1*, *MYOCD*, *MYF6*, *MYF5*, *MYOD1*, *FGF9*, *FGF8*, *MYH11*

**Table 3:** Significantly enriched terms related to the processes of cellular activities.

Term ID	Term	Genes
GO:0055001	muscle cell development	*MYF6*, *ACTA1*, *MYOD1*, *MYH11*
GO:0055002	striated muscle cell development	*MYF6*, *ACTA1*, *MYOD1*
GO:0042692	muscle cell differentiation	*MYOCD*, *MYF6*, *MSTN*, *ACTA1*, *MYOD1*, *MYH11*
GO:0051146	striated muscle cell differentiation	*MYH6*, *MYOCD*, *ACTA1*, *MYOD1*, *MYH11*
GO:0030016	myofibril	*ACTA1*, *MYOD1*, *MYL1*, *MYL4*, *MYOM1*, *MYOM2*
GO:0030239	myofibril assembly	*ACTA1*

### 3.4 KEGG pathway analysis for DEGs

DEGs were annotated through KEGG enrichment analysis in the KEGG database, and the results were shown in Table S5. Among the first 20 pathways with the smallest P values (P<0.05), many are related to the metabolism of amino acids, carbohydrates, and lipids (e.g. alanine, aspartate, glutamate, glycine, serine, and threonine metabolism); the pentose phosphate pathway; and glycolysis/gluconeogenesis (Table 4). Four significantly enriched pathways related to growth and development were identified: the extracellular matrix (ECM)–receptor interaction, focal adhesion, tight junction, and insulin signalling pathways (Table 5). There were 42 DEGs associated with these four pathways.

### 3.5 qPCR validation of DEGs obtained via RNA-Seq

**Figure 3:**
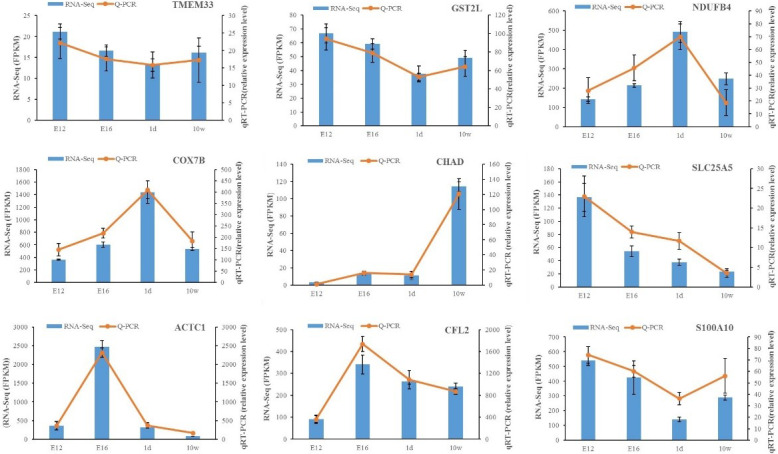
Expression levels of nine DEGs detected by RNA-Seq and validated by qPCR. The results from RNA-Seq are shown in bar graphs, and the FPKM values are shown on the right y axis. The results from qPCR are shown in line graphs, and relative expression values are shown on the left y axis. The data are presented as the mean ± SE.

The expression of DEGs among different comparisons was verified using qPCR. Nine DEGs obtained from RNA-Seq analysis were randomly selected for validation. qPCR was carried out on the same RNA samples used for RNA-Seq. After qPCR analysis of the nine target genes, the qPCR results were consistent with the RNA-Seq results regarding the direction of changes in the expression levels of DEGs, which confirmed the RNA-Seq data (Fig. 3).

## 4 Discussion

Muscle growth and development from the embryonic to the adult stage of an organism consists of a series of exquisitely regulated and orchestrated changes in the expression of genes leading to muscle maturation. In this study, the transcriptome levels of 12 leg muscle tissues at different stages were analysed, and 22 genes were found to be differentially expressed in all comparisons, among which there were three new genes (Novel1117, Novel00182, and Novel01911) and four unannotated genes (ENSGALG00000028710, ENSGALG00000027703, ENSGALG00000040501, and ENSGALG00000043739). In addition, we found that *CD36* (Enciu et al., 2018; Yao et al., 2019), *PHGDH* (Li et al., 2017), *WNT11* (Guo et al., 2018), and *TWIST3* (Kragl et al., 2013) were associated with cell proliferation and that the *ACACB* (Tang, 2015), *ACSL1* (Cao et al., 2018), and *FABP3* (Zhang, 2015) genes play an important role in fat deposition and metabolism. In addition, *Abra* (also known as Stars) is a muscle-specific actin-binding protein capable of stimulating serum response factor (SRF)-dependent transcription through a mechanism involving Ras homolog family member A (RhoA) and actin polymerization (Arai et al., 2002). Abra is involved in human skeletal muscle hypertrophy and atrophy (Lamon et al., 2009). *MAP7* (also known as ensconsin or E-MAP-115) was originally identified based on its association with microtubules (Masson and Kreis, 1993; Bulinski and Bossler, 1994) and was later characterized in non-neuronal cells (Bulinski et al., 1999; Faire et al., 1999). Microtubules are involved in many important cellular processes, including cell division, motility, and changes in cell shape, where microtubule associated proteins (MAPs) bind to and stabilize microtubules (Masson and Kreis, 1995). *MYH1C*, which belongs to the myosin family gene, is the most important structural and functional protein in muscle cells. It is the main component of crude myofilaments and participates in muscle contraction (Zhou et al., 2007; Han et al., 2010). Myosin heavy chain (MyHC) is the main component of the contraction mechanism of skeletal muscle fibres. The expression of the MyHC isotype in the course of injury recovery indicates that MyHC is important for muscle growth and development (Wylie et al., 1995; Zhao and Hoffman, 2004; Parise et al., 2006). Although there are few reports on the function of the *AHSG*, *NTRK2*, *LOC418544*, *LOC417013*, and *PRDH* genes, we speculate that these genes may be related to muscle growth and cell activity.

**Table 4:** Pathways related to the metabolism of amino acids, carbohydrates, and lipids.

Comparisons	Pathways	P value	Genes
E12 vs. E16	Alanine, aspartate, and glutamate metabolism	0.0017	*DDO*, *CPS1*, *GAD2*, *ADSSL1*
	Glycine, serine, and threonine metabolism	0.0380	*LOC418544*, *SRRL*, *GATM*, *GLDC*
E12 vs. 1 d	Glycolysis/gluconeogenesis	0.0111	*PGM1*, *LDHA*, *TPI1*, *FBP2*, *PFKM*, *DLD*, *PDHA1*, *PDHB*, *PGAM1*, *DLAT*, *GAPDH*,
	Alanine, aspartate, and glutamate metabolism	0.0130	*DDO*, *ASNS*, *ADSSL1*, *GOT1*, *GAD2*, *GOT2*, *ALDH5A1*
E12 vs. 10 w	Glycolysis/gluconeogenesis	0.0049	*PGM1*, *FBP2*, *TPI1*, *ADH6*, *PKM*, *ALDOC*, *PGK1*, *PGAM1*, *DLAT*, *GAPDH*, *PFKM*, *LDHA*,
E16 vs. 1 d	Alanine, aspartate, and glutamate metabolism	0.0010	*GOT1*, *GOT2*,
	Glycine, serine, and threonine metabolism	0.0169	*SRRL*, *DMGDH*, *LOC418544*, *CHDH*, *GATM*
E16 vs. 10 w	Glycolysis/gluconeogenesis	0.0094	*PGM1*, *LDHA*, *PFKM*, *PKM*, *ALDOC*, *PGK1*, *FBP2*, *ADH6*
	Pentose phosphate pathway	0.0192	*PGM1*, *PFKM*, *ALDOC*, *FBP2*

**Table 5:** Pathways related to muscle growth and development.

Comparisons	Pathways	P value	Genes
E12 vs. E16	ECM–receptor interaction	0.0001	*LAMB3*, *COMP*, *LAMB4*, *CHAD*, *COL24A1*, *CD36*, *LAMA1*
	Focal adhesion	0.0046	*COMP*, *LAMB3*, *CAV1*, *MYLK2*, *CHAD*, *EGF*, *COL24A1*, *LAMB4*, *MYLKSML*, *LAMA1*, *ACTN2*
	Tight junction	0.0062	*PPP2R2C*, *MYH1B*, *MYH1A*, *MAP3K20*, *CTNNA3*, *CLDN1*, *MYH1C*, *ACTN2*, *PRKCH*, *MYH1E*, *MYH1F*
	Insulin signalling pathway	0.0062	*CALML3*, *PRKAA2*, *PHKA2*, *FBP2*, *PPP1R3A*
E12 vs. 1 d	ECM–receptor interaction	0.0000	*COL1A2*, *COL27A1*, *ITGB8*, *SV2B*, *HMMR*, *COL1A1*, *THBS4*, *CHAD*, *LAMA1*, *CD36*, *THBS2*, *LAMB2*, *SPP1*, *VWF*, *COL24A1*, *LAMB4*, *TNN*, *COL2A1*
	Focal adhesion	0.0286	*COL1A1*, *LAMA1*, *ACTN2*, *RASGRF1*, *COL1A2*, *ITGB8*, *CAV1*, *THBS4*, *CHAD*, *EGF*, *PDGFD*, *CAV2*, *THBS2*, *MYLK4*, *MYLK2*, *TNN*, *SPP1*, *LAMB2*, *COL1A2*, *COL24A1*, *LAMB4*, *MYLKSML*, *PAK5*, *COL27A1*, *COL2A1*, *VWF*
E12 vs. 10 w	ECM–receptor interaction	0.0000	*LAMB4*, *LAMA1*, *COL1A2*, *LAMB3*, *ITGB8*, *COMP*, *HMMR*, *COL1A1*, *THBS4*, *THBS2*, *CD36*, *CHAD*, *SV2B*, *SPP1*, *LAMB2*, *COL1A2*, *VWF*, *COL24A1*, *COL27A1*, *TNN*, *COL2A1*
	Focal adhesion	0.0148	*COL1A1*, *LAMA1*, *RASGRF1*, *COL1A2*, *LAMB3*, *ITGB8*, *CAV1*, *THBS4*, *CHAD*, *EGF*, *PDGFD*, *VWF*, *THBS2*, *MYLK4*, *ACTN2*, *CAV2*, *MYLK2*, *SPP1*, *LAMB2*, *COL1A2*, *COMP*, *RAC2*, *COL24A1*, *LAMB4*, *MYLKSML*, *COL27A1*, *TNN*, *COL2A1*
E16 vs. 1 d	ECM–receptor interaction	0.0013	*CD36*, *COL6A3*, *HMMR*, *COL1A1*, *THBS4*, *VWF*, *COL1A2*, *LAMB4*, *THBS2*
E16 vs. 10 w	ECM–receptor interaction	0.0000	*COL1A2*, *COMP*, *HMMR*, *COL1A1*, *THBS4*, *SV2B*, *LAMB4*, *THBS2*, *CHAD*, *VWF*, *COL2A1*
1d vs. 10w	ECM–receptor interaction	0.0188	*CD36*, *CHAD*, *THBS1*, *COL2A1*,

The differential expression of genes related to growth and development is considered the primary reason for genetic variation in chicken growth and development. Among the DEGs involved in growth and development identified in this study, some were previously reported to be closely related to muscle growth and development. As a regulator of skeletal muscle growth, *MSTN* plays a key role in negatively regulating the growth and development of skeletal muscle and influencing the strength and quality of muscles (Yi et al., 2016; Liu et al., 2016; Rao et al., 2016). *MSTN* can inhibit the activation, proliferation, and differentiation of myogenic satellite cells (McFarland et al., 2006), and delayed differentiation of myoblasts due to a lack of *MSTN* results in significant changes in the morphology of myoblasts (Sato et al., 2006), which indicates that *MSTN* plays an important role in chicken embryogenesis and skeletal muscle development and is one of the important factors regulating muscle development. In this study, the *MSTN* gene was expressed in different developmental stages, and the expression of the *MSTN* gene was high at E16 and 10 w (Table S6), which was consistent with the results of previous studies showing that the *MSTN* gene is mainly expressed in skeletal muscle in the growth period and mature stage (Hu et al., 2004). *MYOD*, *Myf5*, and *Myf6* are members of the myogenic determination factor family, which controls the proliferation and differentiation of muscle cells and is closely related to the number and size of muscle fibres. Therefore, we speculated they played an important role in meat quality and flavour, and the members of this family can control many key regulatory factors in skeletal muscle, either individually or collaboratively (Naidu et al., 1995). In the primary stage of muscle development, *Myf5* is an important factor in the activation of skeletal muscle satellite cells and plays an important role in the differentiation of pre-myoblasts and the proliferation of myoblasts (Sobolewska et al., 2011; Yin et al., 2014). Compared with *Myf5*, the mRNA of *Myf6* appears late, only in the myogenic phase and is expressed in different stages of tubule maturation (Hayes et al., 2003; Kassar-Duchossoy et al., 2004; Kent, 2002). The members of the *MyoD* gene family have different functions during muscle development. The results of using gene knockout to study the function of mouse *MyoD* gene family members showed that as long as the *MyoD* or *Myf5* genes remain intact, they can basically satisfy the requirements for skeletal muscle development (Cooper et al., 1999). If mice with *MyoD* and *Myf5* gene knockout show no muscle formation, there is no subsequent transcriptional process promoted by the *MyoG* gene (Rudnicki et al., 1993). These results suggested that *MyoD* and *Myf5* mainly function in the differentiation and proliferation of myoblast pre-cells and directly or indirectly regulate the expression of other members of this family. *MyoG* and *Myf6* are located downstream of the *MyoD* and *Myf5* genes and mainly function in the secondary stage of muscle development, while *Myf6* mainly controls the further fusion of myotubes into muscle fibres. The results showed that the *Myf5* and *MyoD* genes increased first and then decreased at four different developmental stages, in which *Myf5* gene expression reached its highest level at E16, and the *MyoD* gene showed its highest expression at 1 d; the *Myf5* and *MyoD* genes regulated the proliferation and differentiation of myocytes during embryonic development, which was closely related to the number and size of muscle fibres. These results suggested that *Myf5* plays a regulatory role earlier than *MyoD*. However, the *Myf6* gene was expressed throughout the process of development and showed an upward trend, reaching its highest expression at 10 w. This was related to the role of *Myf6* in maintaining differentiation, which not only promotes differentiation at the embryonic stage but also continues to be expressed in adult mammalian muscle tissue (Li, 2008).

The number of muscle fibres is determined embryonically (Smith, 1963), as myoblasts, originating as somites, migrate to the appropriate site of muscle formation and then proliferate during the process of hyperplasia. These myoblasts subsequently exit the cell cycle, fuse to form multi-nucleated myotubes, and differentiate with the commencement of muscle-specific protein expression. After the actual number of muscle fibres is determined during hyperplasia, skeletal muscle stem cells, which are satellite cells located beneath the basal lamina of the muscle fibres, are activated and fuse with muscle fibres to contribute their nuclei, resulting in a further increase in DNA that directly leads to an increase in protein synthesis. This post-hatch increase in muscle fibre size (hypertrophy) is responsible for the majority of overall muscle mass accretion (Velleman, 2007; Moss, 1968). In this study, GO analysis showed that the identified DEGs were mainly related to the muscle system process, embryo development, muscle organ development, muscle tissue development, and striated muscle tissue development categories as well as muscle cell development and differentiation and striated muscle cell development and differentiation. Many DEGs that are known to affect chicken growth and development are associated with these terms, indicating that chicken growth is a complex process that is influenced by multiple genes and controlled by multiple GO terms.

KEGG analysis identified four pathways related to growth: the ECM–receptor interaction, focal adhesion, tight junction, and insulin signalling pathways, among which the ECM–receptor interaction was the most significantly enriched. ECM is a complex mixture of functional macromolecules. The ECM interacts with cells through cell surface-related elements such as integrin that directly or indirectly control cell activities such as adhesion, migration, differentiation, proliferation, and apoptosis (Thomas et al., 2015). In this study, the DEGs were enriched in ECM–receptor interactions at four developmental stages and in pairwise comparisons, which suggested that the ECM–receptor interaction plays an important role in the regulation of muscle growth and development in Haiyang yellow chickens. Three enriched cell junction-related pathways were screened out in the present study, indicating that pathways involved in maintaining the integrity of tissues might be critical for the early growth of chickens. The insulin signalling pathway was involved in translation initiation, and the efficiency of the translation process directly affects the rate of protein synthesis. Insulin, as a component of this pathway, plays a key role in stimulating glucose transport (Laukkanen et al., 2004; Davis et al., 2008). Recently, studies have shown that ontogenetic changes in the expression of genes in this pathway in skeletal muscle contribute to the developmental decline in protein synthesis (Jiang et al., 2010). Thus, this pathway is considered to be involved in growth, differentiation, and metabolism (Saltiel and Pessin, 2002). In this study, DEGs were significantly enriched in the insulin signalling pathway in the E12 vs. E16 comparison, suggesting that there were major differences between the E12 and E16 embryonic ages in the processes of protein synthesis, glucose metabolism, and cell growth. Focal adhesions are large dynamic protein complexes that are targeted by biochemical and mechanical stimuli from the extracellular environment and can evoke crucial developmental and injury response mechanisms, such as cell growth, movement, and differentiation. Focal adhesions are considered to act as mechanical linkages to ECM (Romer et al., 2006). The expression patterns of DEGs belonging to these two pathways (including *CHAD*, *LAMB4*, *COL2A1*, *LAMA1*, and *COMP*) were very similar, and their enrichment was mainly concentrated around E12, which indicates that there is considerable capacity for satellite cell activities and skeletal muscle development at E12 compared with other stages. The tight junction is a cellular structure that functions as a barrier to restrict the free passage and movement of ions, liquids, proteins, and large solutes through the paracellular pathway (Gonzalez-Mariscal et al., 2008; Citi and Cordenonsi, 1998). This barrier function is essential for the development of multicellular organisms (Shen et al., 2011). Another role of tight junctions has been revealed, showing that they are involved in the control of cell proliferation and gene expression (González-Mariscal et al., 2007). The DEGs were significantly enriched in the tight junction category in the E12 vs. E16 comparison, which suggested that this pathway is essential for the development of multicellular organisms and cell proliferation.

## Supplement

10.5194/aab-64-405-2021-supplement The supplement related to this article is available online at: https://doi.org/10.5194/aab-64-405-2021-supplement.

## Data Availability

Data used in this paper are available upon request.
