# Supplementary material for: Transcriptomic profile of leg muscle during early growth and development in Haiyang yellow chicken
Source: Arch Anim Breed. 2021 Sep 20;64(2):405–16. doi: 10.5194/aab-64-405-2021 (PMC8461557; doi:10.5194/aab-64-405-2021)
Supplement: The supplement related to this article is available online at: https://doi.org/10.5194/aab-64-405-2021-supplement. [file aab-64-405-supplement.zip › aab-64-405-2021-supplement-title-page.pdf]

Supplement of Arch. Anim. Breed., 64, 405–416, 2021
https://doi.org/10.5194/aab-64-405-2021-supplement
© Author(s) 2021. CC BY 4.0 License.

Open Access
Archives Animal Breeding

Supplement of

Transcriptomic profile of leg muscle during early growth and development in Haiyang yellow chicken

Xuemei Yin et al.

Correspondence to: Xuemei Yin (d160079@yzu.edu.cn) and Jinyu Wang (jywang@yzu.edu.cn)

- aab-64-405-2021-supplement-title-page.pdf
- Supplementary Files
  - Table S1.xlsx
  - Table S2.xlsx
  - Table S3.xlsx
  - Table S4.xls
  - Table S5.xls
  - Table S6.xls

The copyright of individual parts of the supplement might differ from the article licence.
